# A Novel Autosomal Dominant Inclusion Body Myopathy Linked to 7q22.1-31.1

**DOI:** 10.1371/journal.pone.0039288

**Published:** 2012-06-18

**Authors:** Yan Lu, Xingang Li, Min Wang, Xin Li, Feng Zhang, Yun Li, Meng Zhang, Yuwei Da, Jun Yu, Jianping Jia

**Affiliations:** 1 Department of Neurology, Capital Medical University, Xuan Wu Hospital, Beijing, People’s Republic of China; 2 Beijing Institute of Genomics, Chinese Academy of Sciences, Beijing,People’s Republic of China; 3 Graduate School of Chinese Academy of Sciences, Beijing, People’s Republic of China; University of Florida, United States of America

## Abstract

We describe a novel autosomal dominant hereditary inclusion body myopathy (HIBM) that clinically mimics limb girdle muscular dystrophy in a Chinese family. We performed a detailed clinical assessment of 36 individuals spanning four generations. The age of onset ranged from the 30s to the 50s. Hip girdle**,** neck flexion and axial muscle weakness were involved at an early stage. This disease progressed slowly, and a shoulder girdle weakness appeared later in the disease course. Muscle biopsies showed necrotic, regenerating, and rimmed vacuolated fibers as well as congophilic inclusions in some of the fibers. Electron micrograph revealed cytoplasmic inclusions of 15–21 nm filaments. A genomewide scan and haplotype analyses were performed using an Illumina Linkage-12 DNA Analysis Kit (average spacing 0.58 cM), which traced the disease to a new locus on chromosome 7q22.1–31.1 with a maximum multi-point LOD score of 3.65. The critical locus for this unique disorder, which is currently referred to as hereditary inclusion body myopathy 4 (HIBM4), spans 8.78 Mb and contains 65 genes. This localization raises the possibility that one of the genes clustered within this region may be involved in this disorder.

## Introduction

Hereditary inclusion body myopathy (HIBM) constitutes a heterogeneous group of disorders,histologically characterized by muscle fibers with rimmed vacuoles and inclusions consisting of filaments with a diameter of 15–21 nm. Currently, five different HIBMs have been reported: one for the autosomal recessive (AR) IBM, which is also known as HIBM2 (MIM #600737), Distal Myopathy with Rimmed Vacuoles (DMRV) or Nonaka Myopathy (MIM #605820) [1], [2], and four for the autosomal dominant (AD) IBMs. The AD IBM includes HIBM1 (MIM #601419), HIBM3 (MIM #605637), HIBM associated with Paget disease of the bone and/or frontotemporal dementia (HIBM-PFD, MIM #167320) and HIBM with early respiratory failure (HIBM-ERF, MIM #607569). HIBM1 is now referred to desmin-related myofibrillar myopathy, which is caused by a mutation in the desmin gene[3]–[5]. HIBM2 is characterized by muscle weakness that initiates within the distal muscles of the lower limbs with relative sparing of the quadriceps [6], [7]. HIBM2 is caused by UDP-N-acetyl-glucosamine 2-epimerase/N-acetylmannosamine kinase (GNE), which is located at 9p13.3 [8], [9]. HIBM3 (MIM #605637) is characterized by congenital joint contractions, external ophthalmoplegia, and a predominant proximal muscle weakness and is caused by a defect in the myosin heavy chain IIa gene located at 17p13.1[10]–[12]. HIBM-PFD, which is located at 9p13, is caused by a defect in the valosin-containing protein gene [13]–[15]. The locus of HIBM-ERF has not yet been identified.

**Figure 1:**
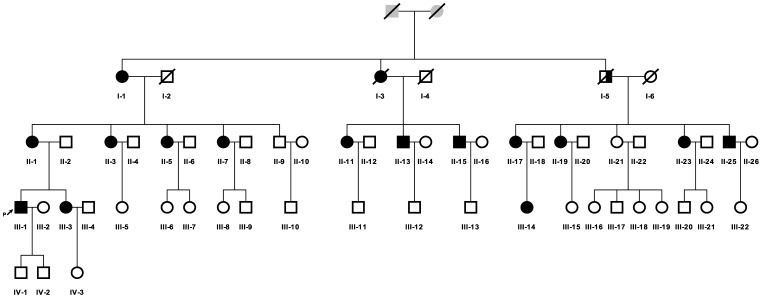
Pedigree of the family. **(**□ male; ○ female; ↗ index patient; ▪ definitely affected; 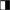 probably affected).

In this study, we describe clinical, myopathological, and genetic findings in a family of Chinese Han decent, which includes 15 cases with IBM. These cases are distributed over three generations with an autosomal dominant pattern of inheritance (figure 1). The GNE mutation was excluded. This disorder is clinically characterized by a late adult-onset weakness that begins in the proximal lower limbs and axial muscles and slowly progress to the proximal upper limbs, which differs from the other AD IBMs. Thus, we performed a genome-wide scan using single nucleotide polymorphism (SNP) markers. Here, we report the identification of a new HIBM locus at 7q22.1-31.1, which we named HIBM4, thus increasing the subtype for HIBM.

## Results

### Pedigree Analysis

The index case developed mild difficulty doing sit-ups and could not jump as high as before at the age of 36. The analysis of his family’s pedigree revealed the existence of 16 affected patients (12 female patients and 4 male patients) distributed in three generations, displaying probably autosomal dominant (AD) inheritance (Figure 1). However, case I-5 did not demonstrate any symptoms when he was suddenly killed in a traffic accident at 40 years of age. According to the pedigree analysis, he was most likely also affected.

### Clinical Features and Course

Our clinical findings for the individual patients are summarized in table 1. All affected individuals had a normal birth and motor development. The age at onset varied from 30s to 50s years and the age at ascertainment ranged from 4 to 83 years in 2009. It appeared that the age at onset of the affected male individuals (range 36–38 years) was earlier than that of the females (range 40–50 years). The initial symptoms were hip girdle and proximal lower limbs involvement in 8 patients. The patients noted difficulty in climbing stairs as well as arising from a squatting position or doing sit-ups. Five patients (patient II-7, 17, 19, 23, III-14) were unaware of limb muscle weakness, but examination revealed weakness of hip flexors. Patient III-3 denied any muscle weakness, however, examination revealed the neck flexor weakness. Accurately, most of the patients demonstrated asymptomatic neck flexor weakness, except patient II-25. The mild shoulder girdle and proximate upper extremity weakness were observed in 5 of the 15 affected individuals. However, 4 of these patients were asymptomatic and the disease course was more than 5 years in all five patients. Distal lower limb weakness was observed in four patients, who were unable to walk on their heels or on their toes (patient I-1, II-1, 3, 13). One patient (Patient I-1) had a distal upper limb weakness. Cranial nerve innervated muscles were spared. In affected muscles, myotatic reflexes were reduced or abolished and no myotonia was detected. The patients showed no inflammatory signs such as pain, swelling or redness. The sensory examination was normal in all patients. Furthermore, muscle atrophy was not prominent in the early stage but was moderate in the advanced stage.

**Table 1:** Clinical findings in 15 affected individuals with muscle weakness.

Case	I-1	II-1	II-3	II-5	II-7	II-11	II-13	II-15	II-17	II-19	II-23	II-25	III-1	III-2	III-14
Sex	F	F	F	F	F	F	M	M	F	F	F	M	M	F	F
Age at examination(yr)	83	62	52	47	45	46	44	41	58	55	46	40	37	34	32
Age at onset(yr)	50s	43	45	43	D	40	37	38	D	D	D	D	36	D	D
First symptoms	ProxLegweak	ProxLegweak	ProxLegweak	ProxLegweak	N	ProxLegweak	ProxLegweak	ProxLegweak	N	N	N	N	ProxLegweak	N	N
Cervical muscle weak	Yes	Yes	Yes	Yes	No	Yes	Yes	Yes	Yes	Yes	Yes	No	Yes	Yes	Yes
Difficulty doing sit-ups	P	P	P	P	P	P	P	P	P	P	P	P	P	N	P
Proximal arm	2–3	4	5	5	5	5	4-	4	4	5	5	5	5	5	5
Distal arm	3	5	5	5	5	5	5	5	5	5	5	5	5	5	5
Proximal leg	2	4	4	4	4	4	4-	3	4	4	5-	5	4	5	5-
Distal leg	0	5-	5-	5	5	5	5-	5	5	5	5	5	5	5	5

F = female; M = male; D = deny to have any muscle weakness; N = no symptom.

The disability slowly progressed over the years and the prognosis was relatively benign. Nineteen years after onset, patient II-1 could climb stairs or arise from a squatting position with the help of her hands. Moreover, patient I-1 could maintain daily life activities without any assistance for two decades after onset; however, she was bedridden due to a fall at the age of 78. She was the only patient who had an ankle contracture, and her ankle flexor/extensor strength was difficult to assess because of her reduced joint mobility. All of the other patients were still able to walk independently and none of them had joint movement range limitations.

### Laboratory Investigations

Laboratory examinations of patient III-1 (index case) and patient II-13 were normal including a complete blood count, electrolytes, fasting glucose, blood urea nitrogen, creatinine, aspartate aminotransferase, alanine aminotransferase, thyroxine, thyroid stimulating hormone, sedimentation rate, and C reactive protein. Electrocardiogram and echocardiogram results were normal for the two patients that were subjected to these examinations within the last 6 months.

**Figure 2:**
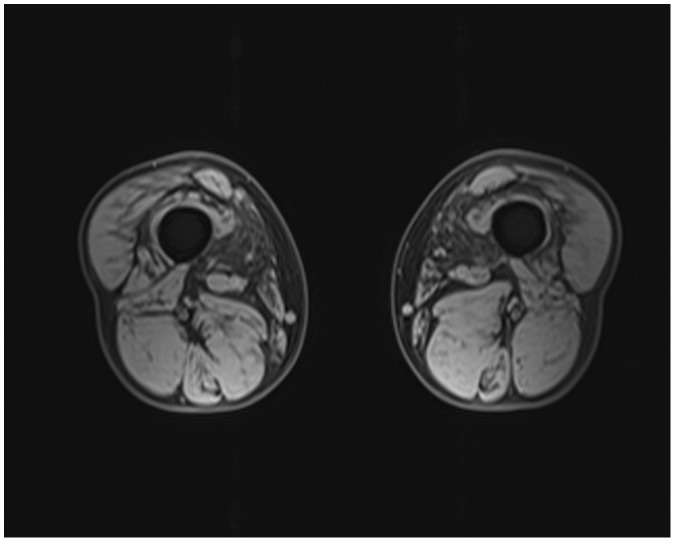
Muscle MRI imaging of the index case showing slight atrophy in the lateral and medial muscles of the thigh.

The serum creatine kinase level was mildly elevated in patient II-13 (377IU/L, normal value was 18-198 IU/L) and patient III-1 (342 IU/L). Needle electromyography revealed a myopathic pattern in patient II-13 and patient III-1 (fibrillation potentials and positive sharp waves, low amplitude, short duration motor unit action potentials demonstrating early recruitment). Furthermore, the upper and lower extremity sensory and motor nerve conduction studies were normal. In addition, a muscle magnetic resonance imaging (MRI) performed at 37 years of age in the index case showed a diffuse involvement of the thigh muscles, with slight involvement in the lateral and medial muscles (Figure 2), compared with the hamstring muscles.

### Muscle Pathology

**Figure 3:**
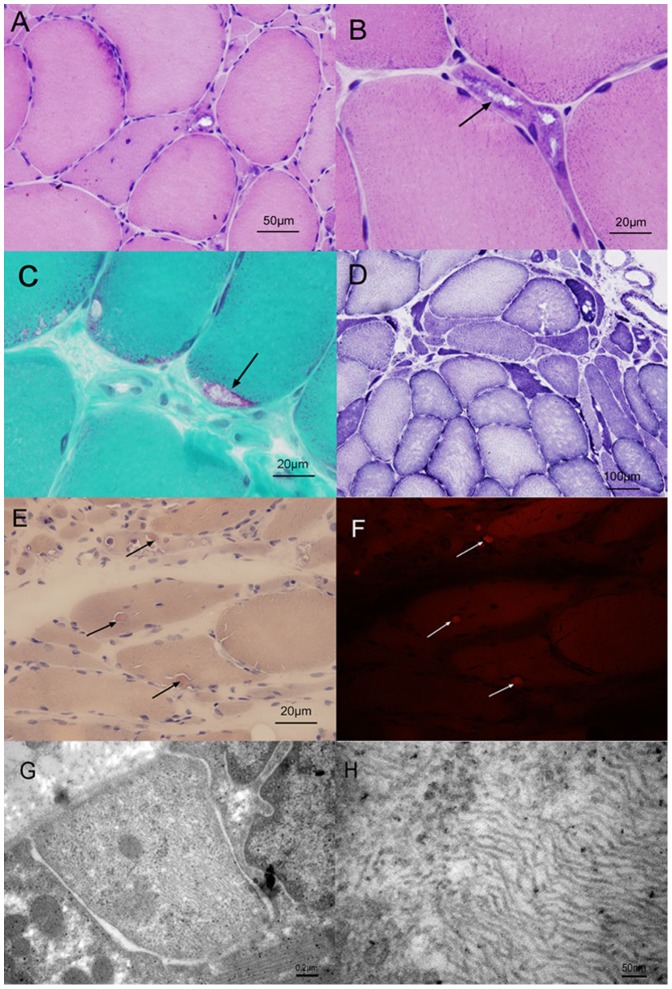
Serial sections of a muscle biopsy obtained from the left quadriceps femoris muscle of the index patient (patient III-1). A, Hematoxylin and eosin-stained cryostat section of muscle showing variations in fiber size and single or grouped atrophic fibers. B and C, Hematoxylin-eosin and modified Gomori trichrome stains demonstrating rimmed vacuoles (arrows). D, NADH dehydrogenase reacted section showing several fibers harbored focal decreases of enzyme activity. E and F, Congo red stained sections viewed under rhodamine optics showing small congophilic deposits in a few fibers (arrows). G and H, Electron micrograph showing cytoplasmic filamentous aggregates in a muscle fiber.

Muscle biopsy from patient III-1 showed myopathic and neuropathic changes with marked fiber size variation, scattered or clusters of small atrophic angulated fibers, occasional degenerating and necrotic fibers (Figure 3A). Inflammatory changes were absent. On cryostat sections, the most prominent finding was the presence of rimmed vacuoles in many atrophic fibers, lined by basophilic granular material on hematoxylin-eosin (H&E) staining (Figure3B) and purple-red in color with the modified Gormori trichrome (MGT) stain (Figure 3C). Several fibers contained cytoplasmic bodies. In NADH dehydrogenase reacted sections, several fibers harbored focal decreases of enzyme activity (Figure 3D). The atrophic fibers are of either histochemical type, some of which overreact for nonspecific esterase, indicate denervation atrophy. A mosaic of type I/II fibers was detected in the ATPases reactions, with no evidence of fiber type grouping. There was no unusual reactivity for CD4 or CD20. Occasional cells were CD8 positive and several cells were CD68 positive. Rare atrophic fibers harbored abnormal accumulations of desmin. Congo red stained sections viewed under rhodamine optics showed small congophilic deposits in a few fibers (Figure 3E, 3F). Occasional fibers showed increased, focal reactivity for alpha B-crystallin. No abnormal reactivity for myotilin was observed. Electron micrograph showed cytoplasmic inclusions of 15–21 nm filaments aggregated in a muscle fiber (Figure 3G, 3H). Muscle biopsy from patient II-13 showed combined neuropathic and myopathic changes, including marked fiber size variation, scattered or clusters of small atrophic angulated fibers, occasional degenerating and necrotic fibers. Inflammatory changes were absent. Rimmed vacuoles were present in some fibers.

### GNE Gene Sequencing

Polymerase chain reactions and analysis of the eleven coding exons (exons 2–12) of GNE were performed but no mutations were detected (data not shown).

### Linkage Analysis and Haplotyping

The unaffected offsprings were not included in the linkage analysis, because a multipoint analysis in MERLIN showed pedigree size and program memory constraints. This would not affect the result of the nonparametric linkage (NPL) analysis which *per se* uses only affected individuals. For the parametric linkage, this would result in a loss of information. However, most of the currently unaffected individuals in the pedigree were under the average age of onset, thus phenotypic mis-specification may be introduced if these individuals were treated as truly “unaffected” in the linkage analysis. Therefore, to ensure the accuracy of the linkage results and to keep MERLIN running smoothly, we excluded the currently unaffected individuals.

**Figure 4:**
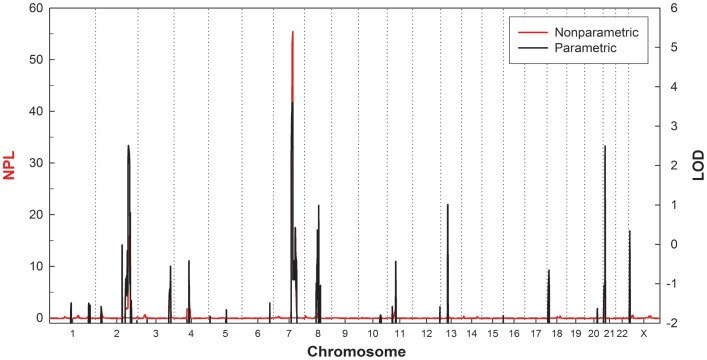
The multipoint parametric and nonparametric linkage scores across the genome. The nonparametric linkage (NPL) scores are shown in red on the left vertical axis, and the parametric LOD scores assuming a dominant inheritance are shown in black on the right vertical axis.

The results of the genome-wide linkage analysis are presented in Figure 4. The prominent linkage signal across the genome was found on chromosome 7q22.1-31.1, with a LOD score of 3.608 and a NPL score of 55.440 (*P<*0.00001). Two other regions with LOD scores greater than 2.0 were observed on chromosome 2 (LOD = 2.512, NPL = 15.89 at 204.2 cM) and chromosome 21 (LOD = 2.50, NPL = 4.87 at 11.83 cM).

**Figure 5:**
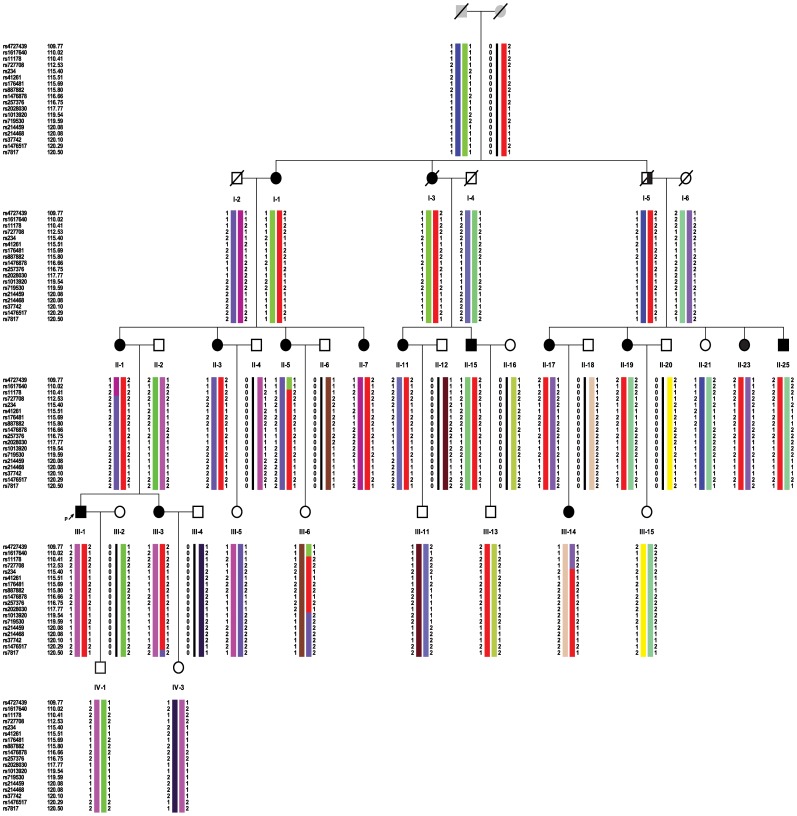
Suggested haplotypes of chromosome 7q22.1-31.1 linked to HIBM in the pedigree. The common region shared by all affected individuals of the pedigree is indicated by the red bars.

Haplotypes were constructed to determine the minimal cosegregating interval on chromosome 7q22.1-31.1 in the affected individuals. The suggested haplotypes of this pedigree are shown in the Figure5. The centromeric boundary of the interval on 7q was defined by a recombination event between the SNP markers rs727708 and rs11234 which was observed in patient III-14. The telomeric boundary of the interval corresponds to a recombination event between rs1476517 and rs7817 in patient III-3. These recombination events defined the susceptibility region on 7q to a 7.97 cM interval (112.53 - 120.50 cM; 103.12–111.90 Mb) between rs727708 and rs7817.

## Discussion

The family reported here demonstrates autosomal dominant trait inheritance based on the following observations: (1) multiple affected members in three generations; (2) both sexes are affected; (3) direct male-to-male transmission (I-5 to II-25); and (4) of the 19 family members over thirty years old at risk for inheriting the disease, fifteen (79%) were affected. However, the most important observation in this family was that the linkage analysis provided strong evidence for linkage at 7q22.1-31.1. Moreover, in this family, there was an increased frequency of the disorder in females (twelve of the thirteen were at-risk females and four of the five were at-risk males). This may reflect the smaller total number of males in the kindred. However, we could not explain exactly why three of the four male patients exhibited more severe weakness clinically than that of the female patients. In some extent, it may be due to performing more exercises before onset (patient III-1 was a national 2-level athlete, and patients II-13 and II-15 had practiced Chinese kung fu since childhood).

The phenotype of this chromosome 7q22.3-31.2 linked autosomal dominant muscle disease includes the onset of symptoms between the ages of 30 and 50 years and a slowly progressive proximal limb and neck flexor muscle weakness that remains benign. Serum CK was mildly elevated and EMG was myopathic. Muscle biopsy from the index patient showed a myopathic and neurogenic pattern and rimmed vacuoles with inclusions of 15–21 nm filaments in the cytoplasma. The progressive proximal limb weakness with rimmed vacuoles in the skeletal muscle is also observed in some autosomal dominant limb-girdle muscular dystrophies such as LGMD1A, LGMD1G and LGMD1D/E [16]–[18]. LGMD1A exhibits a distinctive dysarthric pattern of speech and is caused by mutations in the gene coding myotilin (mapped to 5q31) [19], [20]. However, we did not detect the deposition of myotilin protein in the affected muscles of our patient (the myotilin staining was negative). LGMD1G is associated with progressive fingers and toes flexion limitation that is mapped to 4p21 [17]. These symptoms were not present in our family. Recently, Harms and his colleagues [21] found that the genetic etiology of LGMD1D/E was the DNAJB6 gene mutations on 7q36. Muscle biopsies from 3 individuals in one family showed a chronic myopathy with vacuoles whithout available electron microscopy data in these cases. Given the non-specificity of the presence of rimmed vacuoles, Jongen PJH et al. [22] reviewed 1,600 muscle biopsies for rimmed vacuoles and 750 biopsies for filamentous inclusions. Their results revealed that 3 LGMD patients with RVs with light microscopy, but absence of 1521 nm filamentous inclusions by electron microscopy. However, recently Hackman and colleagues [23] reported five Finnish families with LGMD1D/E linked to 7q36. The muscle biopsies from their patients showed myopathic and/or dystrophic features with rimmed vacuoles varied from a few to abundant. Tubulo-filamentous inclusions were seen in close to autophagic vacuoles and in a few myonuclei. In our index patient, other than only myopathic and/or dystrophic features in LGMD, the prominent features of the muscle biopsy were vacuolated myofibers associated with myopathic and neurogenic pattern which are typical histological features in HIBM [24]. Neurogenic changes can be found in HIBM muscle and include the presence of scattered angulated, atrophic fibers and nuclear clumps. This may be a predominating feature in some biopsies leading to erroneous diagnosis of a primary neurogenic disease. Consistent with above mentioned, the muscle biopsy from patient II-13 showed combined neuropathic and myopathic changes that include marked fiber size variation, scattered or clusters of small atrophic angulated fibers, and occasional degenerating and necrotic fibers. Based on these findings, the patients were considered as HIBM in the family [25].

However, the clinical features of our family members are not compatible with any known autosomal dominant or recessive HIBM. With the exception of the mode of inheritance, the clinical phenotype of this new form of IBM is similar to an atypical adult onset HIBM2/DMRV which is characterized by muscle weakness and atrophy beginning in the distal muscles of the lower limbs with relative sparing of the quadriceps. However, proximal weakness of the lower limbs and absence of distal weakness were also reported in HIBM2 patients [26]. These findings were consistent with the results obtained in our patients. HIBM2/DMRV is autosomal-recessive and caused by mutations in the GNE gene on chromosome 9p13.3. Over the last ten years, there are now over 60 GNE mutations have been described worldwide associated with IBM2/DMRV in patients of different ethnic backgrounds [7]. In this family, the exons 2–12 of the GNE gene were detected by direct sequencing, but no mutation was identified.

A genetic genome-wide linkage allowed us to map the disease locus to a region at 7q22.1-31.1 that spans 8.78-Mb. Forty-two described genes as well as 23 pseudo genes have been reported in this refined linked area. The analysis of these 42 genes showed that LHFPL3, SLC26A4, SLC26A3, NRCAM, DOCK4, DLD, RELN are associated with some congenital diseases or cancers, and COG5, CBLL1, DNAJB9, IMMP2L, PNPLA8, GPR22, PIK3CG, PRKAR2B, NAMPT, RINT1, MLL5 express proteins that may be involved in cellular and structural functions might be good candidates for HIBM4. The known or putative functions of these candidate genes are listed in table 2.

**Table 2:** The known or putative functions of the candidate genes in this refined linked area.

Gene microsatellite marker coded protein	Genomic location(Kbs)	Information
**LHFPL3** lipoma HMGIC fusionpartner-like 3	103969104–104549005	This gene is a member of the lipoma HMGIC fusion partner (LHFP) gene family, which is a subset of the superfamily of tetraspan transmembrane protein encoding genes. Mutations in one LHFP-like gene result in deafness in humans and mice, and a second LHFP-like gene is fused to a high-mobility group gene in a translocation-associated lipoma.
**SLC26A4** solute carrier family 26,member 4	107301080–107358254	Mutations in this gene are associated with Pendred syndrome, the most common form of syndromic deafness, an autosomal-recessive disease.
**SLC26A3** solute carrier family 26,member 3	107405912–107443678	mutations in this gene have been associated with congenital chloride diarrhea
**NRCAM** neuronal cell adhesionmolecule	107788071–108096841	Cell adhesion molecules (CAMs) are members of the immunoglobulin superfamily. Allelic variants of this gene have been associated with autism and addiction vulnerability.
**DOCK4** dedicator of cytokinesis 4	111366164–111846462	This gene is a member of the dedicator of cytokinesis (DOCK) family. Mutations in this gene have been associated with ovarian, prostate, glioma, and colorectal cancers.
**DLD** dihydrolipoamide dehydrogenase	107531586–107561643	Encodes the L protein of the mitochondrial glycine cleavage system. Mutations in this gene have been identified in patients with E3-deficient maple syrup urine disease and lipoamide dehydrogenase deficiency.
**RELN** reelin	103112231–103629963	Encodes a large secreted extracellular matrix protein thought to control cell-cell interactions critical for cell positioning and neuronal migration during brain development. Mutations of this gene are associated with autosomal recessive lissencephaly with cerebellar hypoplasia.
**ORC5** origin recognition complex,subunit 5	103766788–103848463	ORC is a highly conserved six subunit protein complex essential for the initiation of the DNA replication in eukaryotic cells.
**CBLL1** Cbl proto-oncogene, E3 ubiquitin protein ligase-like 1	107384279–107402083	This gene encodes an E3 ubiquitin-ligase for the E-cadherin complex and mediates its ubiquitination, endocytosis, and degradation in the lysosomes.
**DNAJB9** DnaJ (Hsp40) homolog,subfamily B, member 9	108210356–108215294	This gene is a member of the J protein family. J proteins function in many cellular processes by regulating the ATPase activity of 70 kDa heat shock proteins. The encoded protein is localized to the endoplasmic reticulum and is induced by endoplasmic reticulum stress and plays a role in protecting stressed cells from apoptosis.
**IMMP2L** IMP2 inner mitochondrial membrane peptidase-like (S. cerevisiae)	110303110–111202347	Encodes a protein involved in processing the signal peptide sequences used to direct mitochondrial proteins to the mitochondria.
**PNPLA8** patatin-like phospholipasedomain containing 8	108112071–108166638	Encodes a member of the patatin-like phospholipase domain containing protein family. Members of this family are phospholipases which catalyze the cleavage of fatty acids from membrane phospholipids. The product of this gene is a calcium-independent phospholipase.
**GPR22** G protein- coupled receptor 22	107110502–107116125	This gene is a member of the G-protein coupled receptor 1 family and encodes a multi-pass membrane protein.
**PIK3CG** phosphoinositide-3-kinase, catalytic, gamma polypeptide	106505924–106547592	This gene encodes a protein that belongs to the pi3/pi4-kinase family of proteins. The gene product is an important modulator of extracellular signals and maintains the structural and functional integrity of epithelia.
**PRKAR2B** protein kinase, cAMP- dependent,regulatory, type II, beta	106685178–106802256	Encoded one of the regulatory subunits of cAMP-dependent protein kinase. cAMP is a signaling molecule important for a variety of cellular functions.
**NAMPT** nicotinamidephosphoribosyltransferase	105888731–105925638	The protein belongs to the nicotinic acid phosphoribosyltransferase (NAPRTase) family and is thought to be involved in many important biological processes, including metabolism, stress response and aging.
**RINT1** RAD50 inter- actor 1	105172532–105208124	RINT1 may play a role in cell cycle control after DNA damage.
**MLL5** myeloid/lymph- oid ormixed-lineage leukemia 5	104654637–104754532	encodes a protein with an N-terminal PHD zinc finger and a central SET domain. Overexpression of the protein inhibits cell cycle progression.
**SRPK2** SRSF protein kinase 2	104756823–105029341	RNAi-mediated depletion in HeLa cells showed that SRPK2 is essential for cell viability
**LAMB1** laminin, beta 1	107564246- 107643804	Laminins, a family of extracellular matrix glycoproteins, are the major noncollagenous constituent of basement membranes. They have been implicated in a wide variety of biological processes including cell adhesion, differentiation, migration, signaling, neurite outgrowth and metastasis. Laminins are composed of 3 non identical chains: laminin alpha, beta and gamma
**COG5** component of oligomeric golgicomplex 5	106842189–107204959	Encoded one of eight proteins (Cog1-8) which form a Golgi-localized complex (COG) required for normal Golgi morphology and function.

The gene information was obtained from: http://www.ncbi.nlm.nih.gov.

We conclude that this familial disorder is a new variant of HIBM. The genome-wide linkage scan revealed a novel susceptibility region on chromosome 7q22.1-31.1. We propose to classify this AD form of HIBM as HIBM4. Furthermore, exon-trapping studies, and the identification of the gene and gene product responsible for the phenotype in this family will be important for understanding the molecular mechanisms and the pathogenesis of HIBM.

## Materials and Methods

### The Pedigree of the Family

The index case (patient III-1, Figure 1.) was a 37-year-old male, who was referred to our department for progressive weakness in his lower limbs. His mother and maternal grandmother also exhibited similar symptoms. An extended family investigation was performed. We carried out a detailed clinical assessment of 36 individuals spanning four generations. These cases were clinically and neurologically examined by one of the authors (Da Y.W.). The muscle power degree was determined according to the Medical Research Council (MRC) grading scale. Family members were classified as definitely affected if they exhibited symptoms and clinical signs of muscle weakness. The study was conducted after receiving written informed consent from the patients. In addition, this study was approved by the Institutional Ethics Committee of Xuan Wu Hospital, the Capital Medical University.

### Laboratory Tests

Laboratory examinations of the index case and patient II-13 included electrocardiography, echocardiography, electromyography, and routine laboratory tests. Biochemical analysis included serum creatine kinase, electrolytes, fasting glucose, blood urea nitrogen, creatinine, aspartate aminotransferase, alanine aminotransferase, thyroxine, thyroid stimulating hormone, sedimentation rate, and C reactive protein.

### Muscle Imaging

Muscle imaging studies were performed in the index case. Transverse, coronal T1-weighted, T2-weighted and fat-suppressed magnetic resonance imaging (MRI) were performed on a 1.5-T machine (Siemens 1.5T Sonata).

### Myopathological Studies

Muscle biopsy from the quadriceps was performed in the index patient. The sample was snap-frozen in liquid isopentane. Histological and histochemical studies were performed on cryostat sections using hematoxylin-eosin (H&E), modified Gormori trichrome (MGT), NADH-tetrazolium reductase, succinate dehydrogenase, periodic acid–Schiff, nonspecific esterase, Oil red, myofibrillar ATPase, and Congo red. Immunohistochemical analyses were performed to examine the expression of CD4, CD8, CD20, CD68, desmin, myotilin, and alpha B-crystallin (Novocastra). The sections were fixed in acetone and the immunoreactive material was visualized by the ABC system as instruction by manufacturer. Electron microscopic examination was performed using standard techniques. In addition, the muscle biopsy from the triceps was performed in patient II-13 in another hospital (Peking Union Medical College Hospital) in 2007.

### Molecular Tests

Peripheral blood samples from 36 family members were collected. DNA was extracted from whole blood using phenol-chloroform extraction, followed by ethanol precipitation. DNA was diluted in TE to a concentration of 100 ng/µl and stored at 4°C. The entire coding region (exons 2–12) of GNE gene was amplified and sequenced.

### SNP Microarray Genotyping

We genotyped 26 family members using Illumina Infinium HumanLinkage-12 panel (Illumina, San Diego, USA). A genome-wide scan was performed with a total 6,090 single nucleotide polymorphism (SNP) markers within an average gap of 441 kb and 0.58 cM. All reactions were performed according to the manufacturer’s recommendations. Fluorescence signals were scanned using Illumina Bead-station, and a genotype was assigned using the IlluminaBeadStudio Software v3.1.8.

### Linkage Analysis

Mendelian inconsistencies of the genotype data were investigated with Pedcheck for SNPs [27]. All problematic genotypes and markers that were not polymorphic were removed from the data set prior to further processing. After filtering the data, a total of 4,828 informative autosomal SNP markers remained. We used a genetic map provided by Illumina based on the deCODE genetic map for the linkage analysis. Both the parametric and non-parametric linkage analysis were performed using MERLIN [28].The parametric linkage assumed an autosomal dominant model with a risk allele frequency of 0.0001, a penetrance of 0.90 for genotypes with 1 or 2 copies of the risk allele, and a phenocopy rate of 0.00001. We also performed linkage analysis for the X chromosome, assuming a dominant penetrance of 0.90 with MINX [28]. Marker allele frequencies were estimated from the founders of the pedigree via MERLIN. The most probable haplotypes of the pedigree members were also constructed using MERLIN.

## References

[1] Nonaka I, Sunohara N, Ishiura S, Satoyoshi E (1981). Familial distal myopathy with rimmed vacuole and lamellar (myeloid) body formation.. J Neurol Sci.

[2] Nishino I, Noguchi S, Murayama K, Driss A, Sugie K (2002). Distal myopathy with rimmed vacuoles is allelic to hereditary inclusion body myopathy.. Neurology.

[3] Horowitz SH, Schmalbruch H (1994). Autosomal dominant distal myopathy with desmin storage: a clinicopathologic and electrophysiologic study of a large kinship.. Muscle Nerve.

[4] Saavedra-Matiz CA, Chapman NH, Wijsman EM, Horowitz SH, Rosen DR (2000). Linkage of hereditary distal myopathy with desmin accumulation to 2q.. Hum Hered.

[5] Goldfarb LG, Park KY, Cervenakova L, Gorokhova S, Lee HS (1998). Missense mutations in desmin associated with familial cardiac and skeletal myopathy.. Nat Genet.

[6] Argov Z, Yarom R (1984). "Rimmed vacuole myopathy" sparing the quadriceps. A unique disorder in Iranian Jews.. J Neurol Sci.

[7] Jay CM, Levonyak N, Nemunaitis G, Maples PB, Nemunaitis J (2009). Hereditary Inclusion Body Myopathy (HIBM2).. Gene Regul Syst Bio.

[8] Eisenberg I, Hochner H, Shemesh M, Levi T, Potikha T (2001). Physical and transcriptional map of the hereditary inclusion body myopathy locus on chromosome 9p12-p13.. Eur J Hum Genet.

[9] Eisenberg I, Avidan N, Potikha T, Hochner H, Chen M (2001). The UDP-N-acetylglucosamine 2-epimerase/N-acetylmannosamine kinase gene is mutated in recessive hereditary inclusion body myopathy.. Nat Genet.

[10] Darin N, Kyllerman M, Wahlstrom J, Martinsson T, Oldfors A (1998). Autosomal dominant myopathy with congenital joint contractures, ophthalmoplegia, and rimmed vacuoles.. Ann Neurol.

[11] Martinsson T, Darin N, Kyllerman M, Oldfors A, Hallberg B (1999). Dominant hereditary inclusion-body myopathy gene (IBM3) maps to chromosome region 17p13.1.. Am J Hum Genet.

[12] Martinsson T, Oldfors A, Darin N, Berg K, Tajsharghi H (2000). Autosomal dominant myopathy: missense mutation (Glu-706–> Lys) in the myosin heavy chain IIa gene.. Proc Natl Acad Sci U S A.

[13] Weihl CC, Pestronk A, Kimonis VE (2009). Valosin-containing protein disease: inclusion body myopathy with Paget's disease of the bone and fronto-temporal dementia.. Neuromuscul Disord.

[14] Kovach MJ, Waggoner B, Leal SM, Gelber D, Khardori R (2001). Clinical delineation and localization to chromosome 9p13.3-p12 of a unique dominant disorder in four families: hereditary inclusion body myopathy, Paget disease of bone, and frontotemporal dementia.. Mol Genet Metab.

[15] Watts GD, Wymer J, Kovach MJ, Mehta SG, Mumm S (2004). Inclusion body myopathy associated with Paget disease of bone and frontotemporal dementia is caused by mutant valosin-containing protein.. Nat Genet.

[16] Gilchrist JM, Pericak-Vance M, Silverman L, Roses AD (1988). Clinical and genetic investigation in autosomal dominant limb-girdle muscular dystrophy.. Neurology.

[17] Starling A, Kok F, Passos-Bueno MR, Vainzof M, Zatz M (2004). A new form of autosomal dominant limb-girdle muscular dystrophy (LGMD1G) with progressive fingers and toes flexion limitation maps to chromosome 4p21.. Eur J Hum Genet.

[18] Sandell S, Huovinen S, Sarparanta J, Luque H, Raheem O (2010). The enigma of 7q36 linked autosomal dominant limb girdle muscular dystrophy.. J Neurol Neurosurg Psychiatry.

[19] Salmikangas P, Mykkanen OM, Gronholm M, Heiska L, Kere J (1999). Myotilin, a novel sarcomeric protein with two Ig-like domains, is encoded by a candidate gene for limb-girdle muscular dystrophy.. Hum Mol Genet.

[20] Hauser MA, Horrigan SK, Salmikangas P, Torian UM, Viles KD (2000). Myotilin is mutated in limb girdle muscular dystrophy 1A.. Hum Mol Genet.

[21] Harms MB, Sommerville RB, Allred P, Bell S, Ma D (2012). Exome sequencing reveals DNAJB6 mutations in dominantly-inherited myopathy.. Ann Neurol.

[22] Jongen PJ, Ter Laak HJ, Stadhouders AM (1995). Rimmed basophilic vacuoles and filamentous inclusions in neuromuscular disorders.. Neuromuscul Disord.

[23] Hackman P, Sandell S, Sarparanta J, Luque H, Huovinen S (2011). Four new Finnish families with LGMD1D; refinement of the clinical phenotype and the linked 7q36 locus.. Neuromuscul Disord.

[24] Argov Z, D S (2002). Hereditary inclusion body myopathies.. In: G Karpati, (Ed.), Structural and Molecular Basis of Skeletal Muscle Disease.ISN Neuropath Press, Basel, 274–276..

[25] Argov Z, Eisenberg I, Grabov-Nardini G, Sadeh M, Wirguin I (2003). Hereditary inclusion body myopathy: the Middle Eastern genetic cluster.. Neurology.

[26] Motozaki Y, Komai K, Hirohata M, Asaka T, Ono K (2007). Hereditary inclusion body myopathy with a novel mutation in the GNE gene associated with proximal leg weakness and necrotizing myopathy.. Eur J Neurol.

[27] O’Connell JR, Weeks DE (1998). PedCheck: a program for identification of genotype incompatibilities in linkage analysis.. Am J Hum Genet.

[28] Abecasis GR, Cherny SS, Cookson WO, Cardon LR (2002). Merlin–rapid analysis of dense genetic maps using sparse gene flow trees.. Nat Genet.

